# Assessment of Serum Electrolytes and Glycated Hemoglobin Level in Non-diabetic Iron-Deficient Anaemic Patients

**DOI:** 10.7759/cureus.38656

**Published:** 2023-05-07

**Authors:** Shirish Patil, Siddanagouda M Biradar, Renuka Holyachi, Shashidhar Devarmani, Sethu Reddy

**Affiliations:** 1 General Medicine, BLDE (DU) Shri B M Patil Medical College Hospital and Research Centre, Vijayapura, IND; 2 Anaesthesiology, BLDE (DU) Shri B M Patil Medical College Hospital and Research Centre, Vijayapura, IND; 3 Internal Medicine, BLDE (DU) Shri B M Patil Medical College Hospital and Research Centre, Vijayapura, IND

**Keywords:** serum electrolytes, non-diabetics, hba1c, glycated hemoglobin, iron deficiency anaemia

## Abstract

Introduction: The WHO has recognised iron deficiency anaemia (IDA) as the most common nutritional deficiency in the world, with 30% of the population being affected by this condition. The patient's glycemic status during the past three months is shown by the glycated haemoglobin A1C (HbA1c) test. According to several studies, iron deficiency can increase HbA1C levels without affecting blood sugar levels. HbA1C levels of ≥ 6.5% have been approved by the American Diabetes Association (ADA) as a diagnostic indicator for diabetes mellitus (DM). An imbalance in serum electrolyte levels and anaemia have been linked by several studies.

Aim: To analyze the effect of iron deficiency anaemia on HbA1c levels and serum electrolytes in an adult non-diabetic population.

Methods: This was a descriptive cross-sectional study conducted in Shri BM Patil Medical College, Hospital and Research Centre, Vijayapura, Karnataka, India from January 2021 to June 2022. A total of 65 moderate to severe normoglycemic iron deficiency anemia patients between 18 to 75 years were enrolled in the study after fulfilling inclusion and exclusion criteria. A detailed history, clinical and biochemical examination was performed including HbA1c levels. The results were pooled and statistical analyses were performed using Statistical Package for Social Sciences (SPSS) version 20 (IBM Corp., Armonk, NY, USA).

Results: We found elevated HbA1c levels (5.67±1.1%) in non-diabetic iron-deficient anaemia individuals, and elevation was more in women of reproductive age group (30.8%). There was a statistically significant Spearman negative correlation between hemoglobin and HbA1C levels. Also, 16 patients had hyponatremia with a mean haemoglobin (Hb) of 4.8 g/dL and one patient had hyperkalemia with a mean Hb of 3.2 g/dL which was statistically non-significant.

Conclusion: In this study haemoglobin and HbA1c had a statistically significant positive correlation with serum sodium and a negative correlation with serum potassium in moderate to severely iron-deficient anaemic patients, especially females of the reproductive age group.

## Introduction

Over two billion individuals globally, with a mortality rate of almost 800,000 individuals per year, are thought to be anaemic, accounting for one-third of the world's population [1]. The WHO has recognised iron deficiency anaemia (IDA) as the most common nutritional deficiency in the world, with 30% of the population being affected with this condition [2]. Dietary iron insufficiency is the most frequent nutritional ailment in the world, affecting both industrialised and developing countries, regardless of gender or socioeconomic position, and is the leading cause of IDA. An elevated total iron-binding capacity and low serum ferritin or low transferrin saturation characterise the laboratory diagnosis [3].

The N-terminal amino acid of both the ß-chains of haemoglobin undergoes a ketoamine reaction with glucose to form glycohemoglobin [4]. Long-term glycemic management is often evaluated by measuring glycated haemoglobin. Since erythrocytes have an average lifespan of 120 days, when plasma glucose levels are regularly elevated, non-enzymatic glycation of haemoglobin rises. This alteration reflects the glycemic history over the previous two to three months. The HbA1c percentage is unusually high in people with chronic hyperglycemia, and it positively corresponds with metabolic control [5].

Few studies have conclusively linked anaemia to an electrolyte imbalance in the serum as a result of changes in the sodium-potassium adenosine triphosphatase (Na+/K+ ATPase) pump activity that controls intracellular and extracellular cation homeostasis in red blood cells. The serum electrolytes sodium (Na+) and potassium (K+) are necessary for maintaining the characteristic shape of red blood cells (RBCs) and for facilitating gas exchange between RBCs and tissues. They also play a crucial role in nerve transmission and muscle contraction [6]. Na+/K+ ATPase concentration increasing enzyme activity in anaemic patients makes up for the physiological function of enzymes in the cell and the adaptation mechanism for patients with low oxygen levels. Alteration in membrane-bound enzymes have a direct impact on the serum levels of Na+ and K+ [7].

According to previous studies, iron-deficient patients have low sodium levels while potassium levels are high. Lethargy, fatigue, and muscle cramps are examples of moderate symptoms that can result from an alteration in serum electrolyte levels. Severe symptoms include irregular heartbeat, disorientation, convulsions, and even death [8].

Studies on the association between HbA1c and iron deficiency anaemia and how it affects serum electrolytes have produced inconsistent results. The postulated relationship between the two carries major clinical significance since anaemia and serum electrolyte abnormalities are the two public health problems with the greatest prevalence. There hasn't been much research on this topic among Indian people. We decided to conduct the current investigation because of the inconsistent findings of earlier comparable studies in the Indian community, where the incidence of nutritional iron deficiency and electrolyte imbalance is relatively high. 

## Materials and methods

This was a descriptive cross-sectional study conducted in Shri BM Patil Medical College, Hospital and Research Centre, Vijayapura, Karnataka, India from January 2021 to June 2022, after obtaining approval from the institutional ethics committee (IEC/NO-09/2021). A total of 65 moderate to severe iron deficiency anaemic normoglycemic patients between 18 to 75 years were enrolled in the study after obtaining informed consent. Iron deficiency anemia was confirmed via a complete blood count, peripheral smear, serum iron, total iron binding capacity, and serum ferritin levels. The severity of anaemia was classified based on WHO criteria (Table 1). 

**Table 1:** WHO criteria to diagnose severity of anaemia

Hemoglobin (g/dL) levels to diagnose anaemia			
Age	Mild	Moderate	Severe
6 - 59 months of age	10- 10.9	7.0-9.9	<7
5 - 11 years of age	11-11.4	8.0-10.9	<8
12 - 14 years of age	11-11.9	8.0-10.9	<8
Non-pregnant women (≥ 15 years)	11-11.4	8.0-10.9	<8
Pregnant women	10- 10.9	7.0-9.9	<7
Men (≥ 15 years)	11-12.9	8.0-10.9	<8

Formula used to calculate sample size

n = z2 p*q/ d2

Where Z = Z statistic at α level of significance

d2 = Absolute error

p = Proportion rate

q = 100-p

Inclusion criteria

Inclusion criteria were moderate to severe iron deficiency anaemia and age between 18 to 75 years.

Exclusion criteria

Exclusion criteria were Patients with diabetes mellitus, chronic renal failure, hemolytic anaemia, sickle cell anaemia, hemoglobinopathies, chronic alcoholism, endocrinopathies, chronic liver disease, malignancy, recent history of blood transfusion or blood loss, patient on the treatment of IDA in last three months and patients receiving diuretics; Pregnancy, breastfeeding, menorrhagia; Use of drugs: aspirin and antioxidants.

Measurements

The SYSMEX XT-1800i analyser (Kobe, Japan) was used to measure complete blood count by the HGB detector by using the sodium lauryl sulphate (SLS) hemoglobin detection method. The D-10 HbA1C analyser (Bio-Rad, Hercules, CA, USA) utilizes the principles of ion-exchange high-performance liquid chromatography (HPLC) for HbA1C levels. The glucose oxidase/peroxidase method was used for plasma glucose and the 2,4,6-tripyridyls-triazine (TPTZ) for serum iron and total iron binding capacity. Serum ferritin was measured by using the Bio-Rad QuantImune Ferritin IRMA kit based on I-labeled antibody to ferritin.

Statistical analysis

The data obtained were entered in an Excel sheet (Microsoft, Redmond, WA, USA), and statistical analysis was performed using Statistical Package for Social Sciences (SPSS) version 20 (IBM Corp., Armonk, NY, USA). Results are presented as Mean (Median) ± SD, counts, percentages, tables and diagrams. Categorical variables were compared using Chi-square test. Correlation between variables was calculated by Spearman’s correlation. p<0.05 was considered statistically significant. All statistical tests were two tailed.

## Results

The mean age of patients in our study group was 37 years. Our study included 38 females and 27 males. The case distribution among the two sexes is consistent with higher prevalence of iron deficiency anaemia among females of reproductive age group. In the age group of < 30 years iron deficiency anaemic patients were 30.8%, which is higher when compared to other age groups. This is consistent with the finding that iron deficiency anaemia in the reproductive age group is more severe because of negative iron balance which is due to menstrual loss. The mean HbA1c in our patients was 5.67%, minimum and maximum HbA1c levels were 4.8% and 6.7% respectively. Standard deviation was 0.664 and standard error of mean was 0.06. We also observed that 16 patients had hyponatremia with a mean Hb of 4.8 and one patient had hyperkalemia with a mean Hb of 3.2 which was statistically non-significant.

The parameters which impair the HbA1C levels have been measured among all the patients and their mean values were taken. As shown in Table 2, mean value of all the parameters were normal.

**Table 2:** Mean value of parameters HbA1C- Glycated hemoglobin, PCV- Packed cell volume, MCV- Mean corpuscular volume, MCH- Mean corpuscular hemoglobin, MCHC- Mean corpuscular hemoglobin concentration, RDW- Red cell distribution width, TIBC- Total iron binding capacity, FBS- Fasting blood sugar, PPBS- Post prandial blood sugar, ALT- Alanine amino transaminase, AST- Aspartate amino transaminase

Parameters	Mean value
Age	37 years
Hemoglobin	4.9 gm%
HbA1C	5.67%
PCV	16.2%
MCV	68.3 fl
RDW	21.9 ug/dl
Serum Iron	19.1 ug/ml
Serum Ferritin	24.28 ng/dl
TIBC	340 ug/dl
FBS	93 mg/dl
PPBS	124 mg/dl
Blood urea	31 mg/dl
Serum creatinine	0.7 mg/dl
Total bilirubin	0.8 mg/dl
ALT	27.5 U/L
AST	35.5 U/L
Serum albumin	3.7 g/dl

As shown in Table 3, among 65 iron deficiency anaemia patients, 34 patients were in the pre-diabetic range (5.7-6.4) whose mean Hb was 4.8 gm/dl, seven patients were in the diabetic range (>6.4) with mean Hb was 3.1 gm/dl and 24 patients were in the normal range with mean Hb of 5.6 gm/dl (≤5.6).

**Table 3:** Comparison of hemoglobin with HbA1C levels (n)- Number of patients, SD- Standard deviation, HbA1C- Glycated haemoglobin

HbA1c	Hemoglobin (g/dL)			Kruskal-Wallis Test	P Value
	(n)	Mean	SD		
≤ 5.6000	24	5.62	1.023	17.416	0.0001^*^
5.7 - 6.4	34	4.85	1.154		
>6.4001	7	3.12	1.122		
Total	65	4.95	1.310		
*-Statistically Significant					

**Figure 1:**
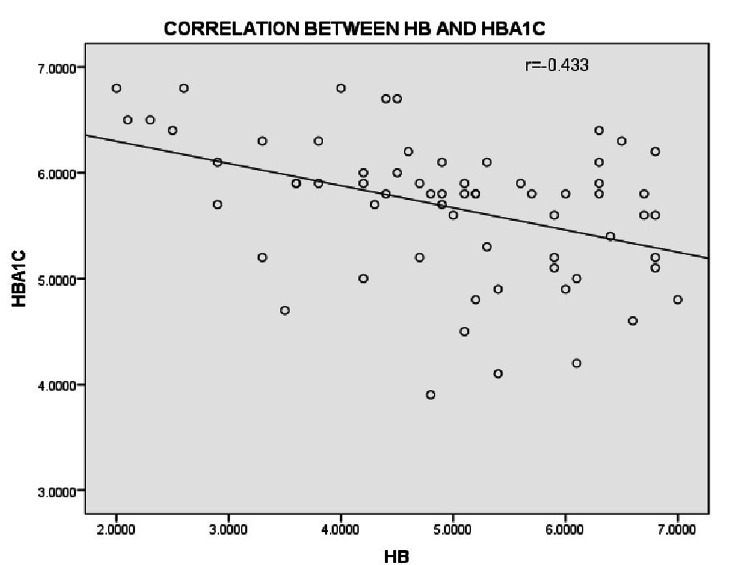
Correlation between hemoglobin and HbA1C levels Hb- Hemoglobin, HbA1C- Glycated hemoglobin.

In our study, as shown in Figure 1, as the haemoglobin levels dropped secondary to iron deficiency the HbA1C levels gradually increased. Also, Table 4 shows mild negative correlation between hemoglobin and HbA1C levels in iron deficiency anaemia which is statistically significant (p = 0.0001).

**Table 4:** Correlation between hemoglobin and HbA1C levels HbA1C- Glycated hemoglobin

Correlation between	Correlation coefficient	P-value	Remark
Hemoglobin and HbA1C	r=-0.433	P=0.0001	Mild Negative correlation. Statistically significant

## Discussion

HbA1C is the most frequently occurring fraction of haemoglobin A1. The glycaemic status for the previous three months is reflected by the HbA1C. In addition to being regarded as the main goal for glycaemic management, it is also a diagnostic criterion according to ADA recommendations [5]. HbA1C values can be impacted by factors other than blood glucose, such as anaemia, which is unrelated to diabetes. Approximately one-third of patients with anaemia have iron deficiency [9].

In addition to a peripheral smear showing microcytic hypochromic blood picture confirming IDA, we also noticed that the haemoglobin and haematocrit were lower in anaemic patients. This is consistent with findings from Barbieri et al. [10].

In this study, we found statistically significant evidence that non-diabetic IDA patients had higher HbA1C levels. This is after excluding participants with additional confounding factors like that are known to alter the HbA1C levels. This finding is consistent with that of Christy et al. who found that iron therapy reduced HbA1C levels, which were noticeably higher in IDA patients [11].

The proposed explanations for higher HbA1C values in IDA were: a) Alteration of quaternary structure of haemoglobin which leads to more rapid glycation of globin chain [12], b) In IDA, decreased hemoglobinization results in older circulating erythrocytes, which raises HbA1C levels [13].

Due to the fact that HbA1C is calculated as a percentage of total Hb, the findings of our study are also in line with those of ElAgouza et al. who found that a decrease in Hb level may result in an increase in the glycated fraction at a fixed glucose level [14]. Moreover, Coban et al. and Kim et al. observed similar findings [15].

Iron plays a crucial role in the body's ability to use and absorb oxygen, which helps to keep RBCs in the physiological state. We found that 16 IDA patients in this study had hyponatremia, while one patient had hyperkalemia. The fact that serum Na+ levels fell is interesting to note. This is consistent with the findings of Rafiq et al., who discovered that patients with IDA have variable serum electrolyte levels [7]. Antwi-Boasiako et al. also found low Na+ levels and high K+ and Cl− in patients with sickle cell anemia [16].

Electrolytes are important for carrying out essential bodily functions like the development and transmission of action potentials in nerves and muscles as well as the preservation of cells' electrical neutrality. Calcium, sodium, potassium, and chloride are essential electrolytes in the body. Normally, the intracellular environment has more K+ ions and the extracellular environment has larger quantities of Na+ ions. In the cell membranes of RBC, there is a protein called Na+/K+ ATPase that keeps this environment stable. It pumps three Na+ ions outside the cell and two K+ ions inside, and is primarily sensitive to changes in pH, membrane integrity, and volume [13]. Studies concluded the significance of Na+/K+ ATPase as an indicator of blood disorders, including anemia.

The most common electrolyte issue among electrolyte imbalance conditions is believed to be hyponatremia. Delirium, nausea, headaches, and vomiting are some of the neurological symptoms that hyponatremia manifests. Arrhythmia is a common symptom of hyperkalemia, which is frequently accompanied by cardiac abnormalities. Hypochloremia is typically brought on by gastrointestinal ion losses [17].

Limitations

Our limitations were the small sample size of the study group, as to generalize the results large group studies are required. Also being a cross-sectional study, it only provides a snapshot of the relationship between iron deficiency anemia, HbA1c, and serum electrolytes at one point in time, but can not determine causality or direction of the relationship.

## Conclusions

This study aimed to investigate the correlation between HbA1c, serum electrolyte imbalance, and IDA among moderate to severe IDA patients between 18 to 75 years of age in India, where the incidence of both nutritional iron deficiency and electrolyte imbalance is high, with inconsistent results from previous studies, and found that hemoglobin and HbA1c had a statistically significant positive correlation with serum sodium and negative correlation with serum potassium in moderate to severely iron deficient anaemic patients, especially females of reproductive age. The findings of this study are largely consistent with the existing literature on the topic.
